# A Novel Mutation in Cse1l Disrupts Brain and Eye Development with Specific Effects on Pax6 Expression

**DOI:** 10.3390/jdb9030027

**Published:** 2021-07-07

**Authors:** Lauren E. Blizzard, Chelsea Menke, Shaili D. Patel, Ronald R. Waclaw, Salil A. Lachke, Rolf W. Stottmann

**Affiliations:** 1 Division of Human Genetics, Cincinnati Children’s Hospital Medical Center, Cincinnati, OH 45229, USA; lauren.blizzard@cchmc.org (L.E.B.); menkeca@miamioh.edu (C.M.); 2 Department of Biological Sciences, University of Delaware, Newark, DE 19716, USA; shailip@udel.edu (S.D.P.); salil@udel.edu (S.A.L.); 3 Division of Experimental Hematology and Cancer Biology, Cincinnati Children’s Hospital Medical Center, Cincinnati, OH 45229, USA; ronald.waclaw@cchmc.org; 4 Division of Developmental Biology, Cincinnati Children’s Hospital Medical Center, Cincinnati, OH 45229, USA; 5 Department of Pediatrics, University of Cincinnati College of Medicine, Cincinnati, OH 45229, USA; 6 Center for Bioinformatics & Computational Biology, University of Delaware, Newark, DE 19716, USA; 7 Institute for Genomic Medicine at Nationwide Children’s Hospital, The Ohio State University College of Medicine, Columbus, OH 43205, USA

**Keywords:** Cse1l, ENU, Pax6, microphthalmia, mutagenesis, anophthalmia, CAS

## Abstract

Forward genetics in the mouse continues to be a useful and unbiased approach to identifying new genes and alleles with previously unappreciated roles in mammalian development and disease. Here, we report a new mouse allele of *Cse1l* that was recovered from an ENU mutagenesis screen. Embryos homozygous for the *anteater* allele of *Cse1l* display a number of variable phenotypes, with craniofacial and ocular malformations being the most obvious. We provide evidence that *Cse1l* is the causal gene through complementation with a novel null allele of *Cse1l* generated by CRISPR-Cas9 editing. While the variability in the *anteater* phenotype was high enough to preclude a detailed molecular analysis, we demonstrate a very penetrant reduction in *Pax6* levels in the developing eye along with significant ocular developmental phenotypes. The eye gene discovery tool iSyTE shows *Cse1l* to be significantly expressed in the lens from early eye development stages in embryos through adulthood. *Cse1l* has not previously been shown to be required for organogenesis as homozygosity for a null allele results in very early lethality. Future detailed studies of *Cse1l* function in craniofacial and neural development will be best served with a conditional allele to circumvent the variable phenotypes we report here. We suggest that human next-generation (whole genome or exome) sequencing studies yielding variants of unknown significance in *CSE1L* could consider these findings as part of variant analysis.

## 1. Introduction

*Chromosome segregation 1-like* (*S. cerevisiae*) (*Cse1l*; also known as cellular apoptosis susceptibility gene CAS or exportin-2) has been linked to nuclear transport, cell cycle maintenance, cell proliferation, apoptosis, and many other cellular functions [1,2]. Nuclear transport is an integral component of many cellular processes and involves several specific transport proteins. Cargo tagged by a classical nuclear localization signal is discriminately bound by an importin-α protein which then forms a heterotrimer with importin-β proteins to be conveyed through the nuclear pore complex [3,4]. Once inside the nucleus, RAN-GTP binds to importin-β, dismantling the heterotrimer and releasing the cargo [5,6]. Importin-α proteins must then be returned to the cytosol to be reused in the next transport cycle. The *CSE1L* protein functions as a re-exporter of importin-α, ensuring its continued availability for nuclear import [7]. Under a variety of cellular stress conditions, importin-α can be imported into the nucleus in the absence of RAN and importin-β. In these conditions, the consequent disturbance in the Ran gradient results in the loss of CSE1L-mediated export of importin-α which initiates a blockage of nuclear import and accumulation of importin-α in the nucleus [8]. This accumulation has been implicated in the expression of factors inducing non-apoptotic cell death [9]. *CSE1L* protein has been shown to localize to microtubule structures in some cells and to play an important role in the G1 cell cycle checkpoint, spindle formation, and chromosome alignment and segregation [2,10]. Lee and colleagues have demonstrated that *Cse1l* interacts with the Ras/Raf/MEK/ERK pathway, as well as acting on the cAMP/PKA pathway via the regulation of CREB and MITF, establishing *Cse1l* as a link between the two signaling networks [11]. CSE1L is a microvesicle membrane protein involved in the formation, and possibly maintenance, of microvesicles induced by Ras activity [12]. *Cse1l* plays a role in maintaining chromosomal stability and DNA repair by regulating RAD51-mediated homologous recombination [13]. Along with other proteins, CSE1L facilitates the formation of the apoptosome [14]. The misregulation of *Cse1l* has been associated with a wide variety of cancers, and it is often utilized as a prognostic marker of tumor grade and pathogenicity. Interestingly, *CSE1L* has the capacity to directly affect transcription of *p53* by association with its promoter [15]. It has been shown that the nuclear transport mechanism requiring CSE1L demonstrates distinct cargo specificity, and this selectivity may indirectly contribute to some epigenetic control within the cell [16,17].

The broad expression of *Cse1l* is mostly correlated with proliferating cells [2,18]. *Cse1l* has been implicated in trophoblast and placenta development via regulation of proliferation and apoptosis [19]. In a study considering endometriosis progression, the downregulation of *Cse1l* was found to affect β-catenin signaling in complex with other interactors, impacting the epithelial to mesenchymal transition [20]. While it has long been known that *Cse1l* is required for early development, it has been difficult to study its mechanism due to the very early lethality of null mice [21]. Here, we present a hypomorphic mutant allele of *Cse1l* discovered in a forward genetic screen which displays a variable range of neural and ocular phenotypes including microphthalmia and ventral telencephalic defects. Our *Cse1l* mouse model presents an invaluable tool to demonstrate a pleitropic role for *Cse1l* in embryonic development.

## 2. Materials and Methods

### 2.1. Animal Husbandry

All animals were maintained through a protocol approved by the Cincinnati Children’s Hospital Medical Center IACUC committee (IACUC2019-0068). Mice were housed in a vivarium with a 12 h light cycle, with food and water ad libitum. Mice to be dissected were euthanized with isoflurane and subsequent cervical dislocation. Genotyping was performed with the following primers: *Cse1l^ant^* F: TTTGTGCCAAGAAGTGTGATG, R: TCAGCAGAGCACAGTCAACA; *Cse1l^null^* F: AGATTCAGAGTCATGGAGCTCA, R: CAATCAGTCAAGGAACAAAGCC; *Cse1l^ant^* TaqMan SNP Genotyping custom probe ID AHMSY60.

### 2.2. ENU Mutagenesis

ENU mutagenesis was performed as previously described on C57BL/6J males [22,23].

### 2.3. Cse1l^null^ CRISPR Design

CRISPR transgenesis was performed with the Cincinnati Children’s Transgenic and Genomic Editing Core. A single guide RNA was used (GATCCTGCCATTAGACGGCC). Genotyping primers were separately designed to detect a large deletion (F: GTTACTTACTCACTTTCTCCTCAGA R: GAATCAATCAGCTGGGCAGAG) and a smaller deletion (F: AGATTCAGAGTCATGGAGCTCA, R: CAATCAGTCAAGGAACAAAGCC). Founders were screened by PCR and Sanger sequence analysis.

### 2.4. Weight

Mice were weighed individually on postnatal day 28 using a standard metric balance.

### 2.5. Histology

Embryos were dissected, fixed in Bouin’s fixative for 48 h, washed in 70% EtOH, and dehydrated and embedded in paraffin by the CCHMC Pathology Core. Blocks were sectioned by microtome at 10 μm, then sections were placed on SuperFrost slides (Cardinal Health, Dublin, OH, USA), baked >1 h, and stained with hematoxylin and eosin using standard methods. All histological and immunohistochemical studies are performed on at least three animal pairs.

### 2.6. Skeletal Preparations

Embryos were dissected at E17.5–E18.5 and frozen. Skin and fat were removed from the embryos which were then fixed in 95% ethanol for 2–5 days. The skeletons were stained with Alizarin-Red and Alcian-Blue (SIGMA-Aldrich, St. Louis, MO, USA) and cleared with potassium hydroxide using standard procedures.

### 2.7. Exome Analysis

Mouse exome analysis was performed at the CCHMC DNA core. Pooled samples were analyzed as a group to find shared homozygous variants as well as individually for each animal. Each variant is annotated based on predicted consequences for the encoded protein. “High-” impact variants are those which are clearly and obviously disruptive to the protein. This would include frame-shift, stop gain variants and disruptions of canonical splice sites. “Moderate-” impact variants include non-synonymous substitutions and in-frame insertions/deletions. Details of analysis are in Supplementary Table S1.

### 2.8. RNA-Seq

E10.5 embryo heads were dissected, snap frozen, and stored at −80 °C. A total of 3 WT and 3 *anteater* mutant heads were each pooled to create one pooled sample of each genotype. RNA was isolated, and pooled samples were each used for paired end bulk RNA sequencing (BGI Americas, Cambridge, MA, USA). Analysis was performed by BGI RNA Sequencing services which includes a proprietary analysis pipeline.

### 2.9. RNAscope

Embryos were dissected at ages E10.5, E12.5, and E14.5 and were fixed in formalin for 16–24 h. The tissue was washed in PBS, then dehydrated and embedded in paraffin by the CCHMC Pathology Core. Paraffin blocks were sectioned on the microtome at 5 μm, placed on SuperFrost slides, then baked at 60 °C for 1 h. Target retrieval steps outlined in the manual assay protocol were followed based on recommendations for brain tissue, then slides were dried at room temperature overnight. Hybridization and amplification steps were performed using the HybEZ oven (Advanced Cell Diagnostics, Newark, CA, USA, ACD)set at 40 °C. Manual assay protocol from was followed using RNAscope Multiplex Fluorescent Reagent Kit V2 (323100), TSA Cyanine 3 Fluorophores (NEL744001KT) at 1:750, and a C1 *Cse1l* probe made to order (Catalog # 591691). All reagents were purchased from Advanced Cell Diagnostics, ACD).

### 2.10. Immunofluorescence (Ascl1, Gsx2, Nkx2.1, Olig2, Pax6)

E12.5 and E14.5 embryo heads were dissected, fixed in 4% PFA overnight, equilibrated in 30% sucrose for 48 h, cryo-embedded in OCT, then sectioned by cryostat at 10 μm. Antigen retrieval was performed with 1% citrate buffer, then sections were incubated in primary antibodies overnight at 4 C: rabbit anti-Pax6 (MBL International, Woburn, MA, USA #PD022, 1:500), goat anti-OLIG2 (R&D Systems, Minneapolis, MN, USA 1:4000), rabbit anti-NKX2.1 (Seven Hills BioReagents, Cincinnati, OH, USA, 1:2000), rabbit anti-GSX2 (Campbell Lab, 1:300 [24]), and rabbit anti-ASCL1 (AbCam, Cambridge, UK 1:400). Sections were incubated for 3 h with Alexafluor 488-conjugated goat anti-rabbit (ThermoFisher Scientific, Waltham, MA, USA #A11008, 1:200) or Alexafluor 488-conjugated donkey anti-goat (AbCam #ab150129, 1:200), counterstained with DAPI, and sealed with ProLong Gold (Invitrogen/ ThermoFisher). Sections were imaged on the Nikon C2 703 confocal microscope.

### 2.11. Immunofluorescence (PHH3, CC3, Tbr2)

Embryo heads were dissected at E12.5 and E14.5, fixed in 4% PFA overnight, equilibrated in 30% sucrose for 48 h, cryo-embedded in OCT, then sectioned by cryostat at 10 μm. Antigen retrieval for pHH3, CC3, was performed with 10% citrate buffer; antigen retrieval for Tbr2 was performed with antigen unmasking solution (Vector Labs, Burlingame, CA, USA). Sections were blocked with 4% normal goat serum in PBST and incubated in primary antibodies overnight at 4 °C: rabbit anti-pHH3 (Sigma #H0412, 1:500), rabbit ant-CC3 (Cell Signaling, Danvers, MA, USA #9661 S, 1:300), and rabbit anti-TBR2 (AbCam #ab31940, 1:200). Sections were incubated for 1 h with Alexafluor 488-conjugated goat anti-rabbit (Thermo #A11008, 1:500), counterstained with DAPI, and sealed with ProLong Gold (Invitrogen). Sections were imaged on the Nikon C2 703 confocal microscope (Nikon Instruments, Melville, NY, USA).

### 2.12. Immunofluorescence (Lhx2, Pax6)

For Pax6 and Lhx2 immunostaining, mouse embryo heads were dissected at stage E10.5, fixed in 4% PFA overnight, equilibrated in 30% sucrose for 48 h, cryo-embedded in OCT, then sectioned by cryostat at 10 mm. For Pax6 immunostaining, sections were thawed and subjected to blocking buffer (2% Bovine Serum Albumin (BSA, Sigma, #A2153-50G), 0.3% TritonX-100 (Fisher Scientific, #NC1365296) in 1X TBS (Tris Buffer Saline)) for 1 h at room temperature followed by permeabilization in 0.05% TritonX-100 in 1X PBS (Phosphate Buffer Saline, Fisher scientific BP243820) for 5 min. Sections were incubated with Pax6 primary antibody (MilliporeSigma, Burlington, MA, USA, #AB2237, raised in Rabbit) in blocking buffer (dilution: 1:150) at 4 °C for 18 h. This was followed by three washes (10 min each) performed in 0.3% TritonX-100 in a 1X TBS solution. Sections were then incubated for 1.5 h at room temperature with Alexafluor 568-conjugated goat anti-rabbit (Thermo Fisher Scientific, #A-11011) and DAPI (1:1000) (Thermo Fisher Scientific, #D21490). This was followed by three washes (10 min each) performed in 0.3% TritonX-100 in a 1X TBS solution. Mounting media was applied and slides were sealed with coverslip and nail polish and visualized by confocal microscopy (Zeiss LSM880 Confocal microscope, Zeiss International, Oberkochen, Germany). For Lhx2 immunostaining, sections were thawed and subjected to an additional fixation step in ice cold 100% 1:1 acetone:methanol for 20 min. Sections were permeabilized in 0.3% TritonX-100 for 10 min in 1X PBS. Sections were then blocked in blocking buffer (10% goat serum (Jackson ImmunoResearch, West Grove, PA, USA, #005-000-121), 0.3% TritonX-100 in a 1X TBS) for 1 h at room temperature. This was followed by incubation with Lhx2 primary antibody (Abcam, #ab184337, raised in Rabbit) in blocking buffer (1:100) at 4 °C for 18 h. This was followed by three washes (10 min. each) performed in 0.3% TritonX-100 in a 1X TBS solution. Sections were then incubated for 1.5 h at room temperature with Alexafluor 568-conjugated goat anti-rabbit (Thermo Fisher Scientific, #A-11011) and DAPI (1:1000) (Thermo Fisher Scientific, #D21490). This was followed by three washes (10 min. each) performed in 0.3% TritonX-100 in a 1X TBS solution. Mounting media was applied and slides were sealed with coverslip and nail polish and visualized by confocal microscopy (Zeiss LSM880 Confocal microscope).

### 2.13. Immunofluorescence Quantification

Nikon Elements software (v 5.21) was used to quantify various aspects of pHH3, Tbr2, CC3, and Pax6 immunofluorescence images. For pHH3, the VZ was delineated as the region of interest, and positive cells within that region were counted. For Tbr2 and CC3, the entire cortex was delineated as the region of interest, and positive cells within that region were counted. Pax6 expression was quantified by delineating the entire cortex as the region of interest, and positive cells within the region were counted. Pax6 expression relative to cortex thickness was quantified by designating linear distances at three regions of the cortex for each image. Pax6 expression area was quantified by designating linear distances spanning the Pax6-positive region at three locations per image.

### 2.14. Western Immunoblotting

E14.5 embryos were microdissected to isolate brain, eyes, and face (frontonasal, maxillary, and mandibular prominences). Tissues were snap-frozen and stored at −80 °C. Tissues were lysed with RIPA buffer with protease inhibitor. BCA assay was performed to determine protein concentration. Protein was loaded into a 4–12% Tris-glycine gel. Protein was transferred to a PVDF membrane, blocked in Intercept blocking buffer, and incubated overnight at 4 C with rabbit anti-CSE1l (CAS) (AbCam #151546, 1:1000) and mouse anti-Tubulin (Sigma #T6199, 1:1000) antibodies. Membranes were washed and incubated for 1 h in goat anti-rabbit IRDye 800CW (LICOR #926-32211, 1:15,000) and goat anti-mouse IRDye 680 Rd (LICOR, #926-68070, 1:15,000) antibodies. Blots were visualized on a LICOR Odyssey imaging system. Relative protein concentration was determined by normalizing Cse1l signal to Tubulin signal in Image Studio Lite Ver 5.2.

## 3. Results

### 3.1. Anteater Mutants Display Variable, Incompletely Penetrant Organogenesis Phenotypes

**Figure 1:**
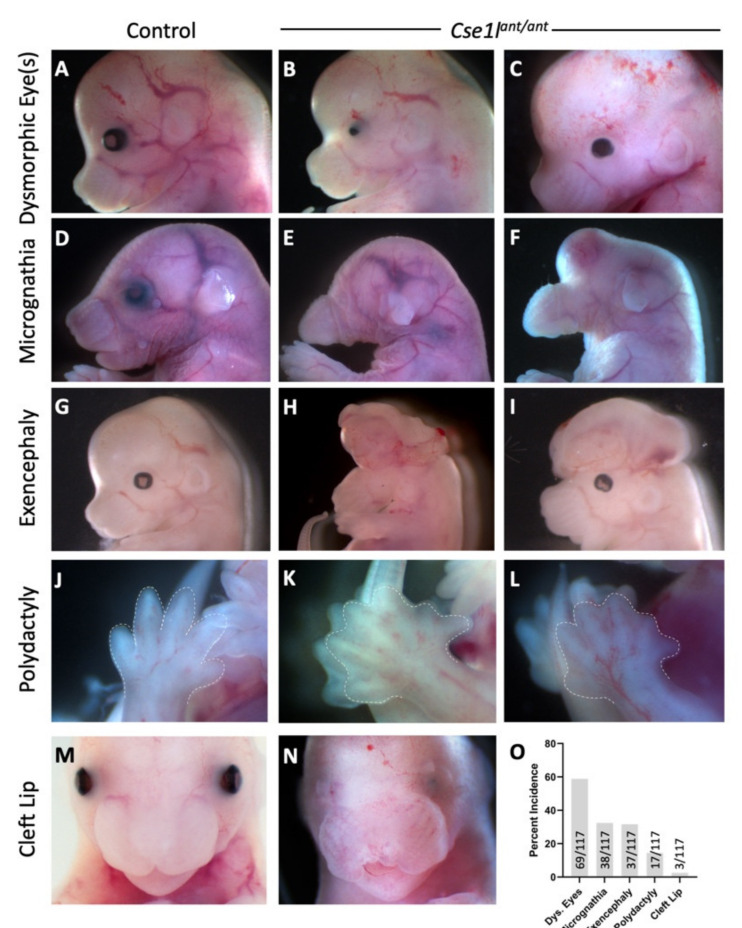
*Cse1l^ant/ant^* mutants display variable, incompletely penetrant phenotypes. (**A**,**D**,**G**,**J**,**M**) Control animals. (**B**,**C**,**E**,**F**,**H**,**I**,**K**,**L**,**N**) *Cse1l^ant/ant^* mutants display a range of dysmorphic eye features, (**B**,**C**) micrognathia and agnathia, (**E**,**F**) exencephaly, (**H**,**I**) polydactyly (**K**,**L**), and cleft lip (**N**); (**O**) Incidence of phenotypes over 117 mutants collected.

We recently continued a mouse forward genetic ENU mutagenesis screen to identify novel alleles important for mammalian organogenesis with a particular emphasis on the developing craniofacial structures. While screening for phenotypes at embryonic day (E) 18.5, we identified a line with a number of notable phenotypes. These were variable and included ocular malformations ranging from microphthalmia (Figure 1B,C) to coloboma (Figure 1I), shortened to nearly absent mandible (Figure 1E,F), exencephaly (Figure 1H,I), polydactyly (Figure 1K,L), and cleft lip (Figure 1N). The most common phenotype was an ocular malformation with decreasing incidence of micrognathia, exencephaly, polydactyly, and cleft lip (Figure 1O). Based on the resemblance of mutants with the most severe phenotypes to the eponymous land mammal, this mutant allele was named *anteater*.

### 3.2. The Anteater Phenotype Is Caused by a Variant in Cse1l

**Figure 2:**
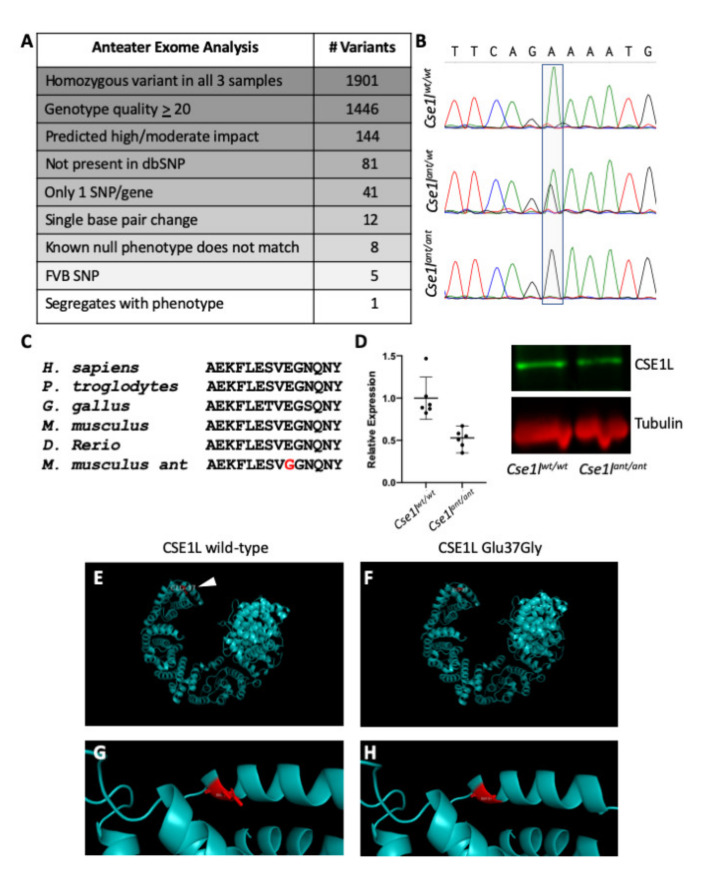
The *anteater* mutant is an allele of *Cse1l.* (**A**) Exome filtering revealed *Cse1l* as a candidate gene for the *anteater* phenotype. (**B**) Sanger sequence of the *anteater* allele. (**C**) CSE1L is a highly-conserved protein: the *anteater* variant is highlighted in red. (**D**) Protein expression was decreased but not absent in *Cse1l^ant/ant^* brains; data are graphed as protein expression relative to the wild type (unpaired *t* test *p* = 0.0012; line indicates mean value +/−95% confidence interval). (**E**–**H**) Predicted protein structure of CSE1L with the position of the variant amino acid highlighted in red.

We performed exome sequencing on three phenotypic *anteater* mutants to identify the causal ENU allele. From an initial list of 26,869 variants in the *anteater* mutants, we filtered for variants which (1) were homozygous in the pooled sample, (2) had a genotype quality score higher than or equal to 20, (3) were predicted to have a “high” or “moderate” effect on the protein, (4) were not present in the dbSNP database (and were thus known strain polymorphisms), and (5) were a single base pair change (known mechanism of ENU mutagenesis). We then further excluded variants in genes for which a null allele was reported and did not phenocopy the *anteater* mutants, or those that were determined to represent a strain-specific polymorphism not recorded in dbSNP. This left five variants, only one of which segregated completely with *anteater* mutants as a homozygote (Figure 2A). The candidate variant is in the *chromosome segregation 1-like (S. cerevisiae) (Cse1l)* gene (chr2: 166,761,506 Mb; A > G). Sanger sequencing confirmed this sequence change as a heterozygote in carriers and homozygous for the alternate allele in mutants (Figure 2B). The missense variant alters the coding of CSE1L protein at a highly conserved portion of the sequence from an acidic, charged glutamate to a hydrophobic glycine (NP_076054 Glu37Gly; Figure 2C). Based on the non-synonymous nature of this variant and conservation of the protein, this was judged to be of “moderate” impact (Figure 2A). Western immunoblotting of tissue isolated from the heads of *anteater* mutant and wild-type E14.5 embryos (Figure 2D) showed an approximate 50% decrease in CSE1L protein levels in mutant tissue as compared to controls (Figure 2D). No obvious changes in protein structure were observed when the predicted protein structure of *Cse1l* was compared with the predicted structure of the *anteater* mutant protein (Figure 2E–H).

We next performed a genetic complementation test to further address the hypothesis that *anteater* is an allele of *Cse1l*. We first used CRISPR-Cas9 genome editing to create a null allele. Mosaic founders were mated and produced *Cse1l^CRISPR /wt^* offspring with a gene modification creating an 8 bp deletion and 4 bp insertion in *Cse1l* (Supplementary Figure S1B). This is predicted to code for a CSE1L protein with 24 appropriate amino acids followed by eleven nonsense residues and a premature stop codon (Supplementary Figure S1B; NP_076054 A24LSQVKMALFLDX). Consistent with previous reports, when we intercrossed these *Cse1l^CRISPR /wt^* mice we found no survival of CRISPR *Cse1l* homozygous null embryos at weaning (Supplementary Figure S1C). We concluded from this that the *Cse1l^CRISPR^* allele is a null allele with a requirement for survival similar to the previously published allele. We then mated *Cse1l^ant/wt^* heterozygous mice with *Cse1l^CRISPR /wt^* mice. None of the offspring at weaning were *Cse1l^CRISPR/ant^* mutants (Supplementary Figure S1D). Of the 42 embryos genotyped between E14.5 and E18.5, none were found to be carriers for both CRISPR *Cse1l* and *anteater* (Supplementary Figure S1D). Thus, the *anteater* allele fails to complement the CRISPR *Cse1l* null allele. We therefore conclude that *anteater* is a hypomorphic allele of *Cse1l* as the mutants survive to organogenesis stages.

### 3.3. Cse1l Expression

**Figure 3:**
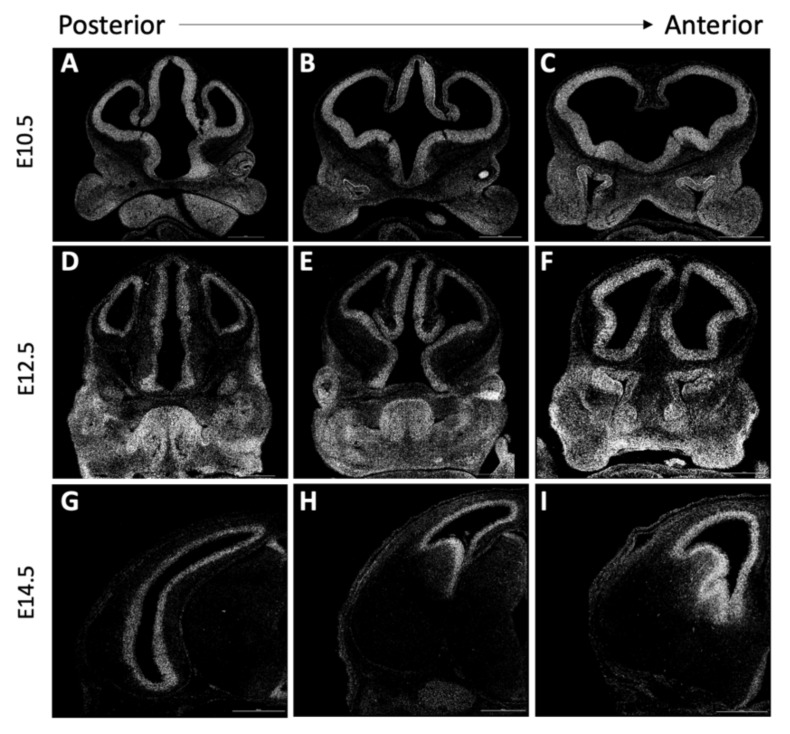
RNAscope shows wild-type *Cse1l* expression is localized to cortex and specific regions of the face and eyes**.** (**A–F**) RNA expression of *Cse1l* in wild-type embryos is widespread throughout the brain and face, and in later stages (**G**–**I**), becomes more localized around the lateral ventricles.

As *Cse1l* expression has not been well-described in the developing mouse embryo, we characterized wild-type *Cse1l* gene expression using single-molecule RNAscope in situ RNA hybridization at embryonic stages E10.5, E12.5, and E14.5 with a particular focus on structures of the head (Figure 3). At all ages analyzed, *Cse1l* expression was quite high in all the forebrain regions examined along the anterior to posterior axis. By E12.5, we observed the expression to be particularly enriched in the ventricular zone. Expression was also evident in the tongue, eyes, mandibular arch, nasal pits, and the future nasal epithelium. Diffuse facial and eye expression of *Cse1l* was noted at E10.5 but became stronger and more regionally defined at E12.5. A significant portion of this expression is consistent with being part of the migrating neural crest cell population. This neural crest expression is also consistent with many of the phenotypes we observe in *anteater* mutants (Figure 1). iSyTE analysis [25,26,27] shows robust *Cse1l* expression in the mouse lens during development and at adult stages (Figure S2). By E14.5, discrete regions of *Cse1l* expression were detected in the tooth buds, salivary glands, and tongue (data not shown). Thus, the embryonic craniofacial, ocular and neural expression of *Cse1l* is consistent with the tissues affected in the *anteater* mutants.

### 3.4. Anteater Mutants Do Not Survive Past Birth and Have a Variable Phenotype

**Figure 4:**
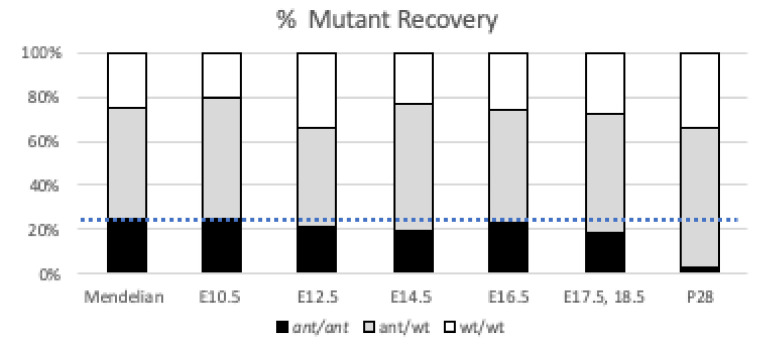
Survival of *Cse1l^ant/ant^* mutants. Percent of mutants recovered from all embryos at each stage analyzed with 25% Mendelian expectations show with blue dotted line.

We noted a nearly complete lack of homozygous *anteater* mutants at weaning (Figure 4, Table 1). Those few mutants that did survive were much smaller than their wild-type and heterozygote littermates but behaved normally (Supplementary Figure S3). Upon dissection, the brain size and structure of these adult mutants appeared normal (Figure S3); however, unilateral microphthalmia was apparent in at least one animal (Supplementary Figure S3). Upon comparing the skulls of the few P28 mice that survived, we discovered that the *anteater* skull was much smaller and at least one animal displayed unilateral dysmorphia in the regions of the nasal bridge and eye socket (Supplementary Figure S3).

The phenotypes we observed at E18.5 (Figure 1) are consistent with perinatal lethality, but we performed dissections at a range of embryonic stages to assess survival through organogenesis and saw near Mendelian ratios of *Cse1l^ant/ant^* homozygous mutant throughout development with a recovery of 73–96% of expected numbers of mutants (Figure 4). Our analysis indicates slightly more statistically robust deviations from Mendelian expectations at E14.5 which we attribute to the significantly large sample size (*n* = 216) as we also targeted this age for molecular analyses. Taken together, these data suggest to us the *Cse1l^ant/ant^* homozygous mutants are slightly less viable than control littermates during embryonic development and almost completely absent by weaning ages.

**Figure 5:**
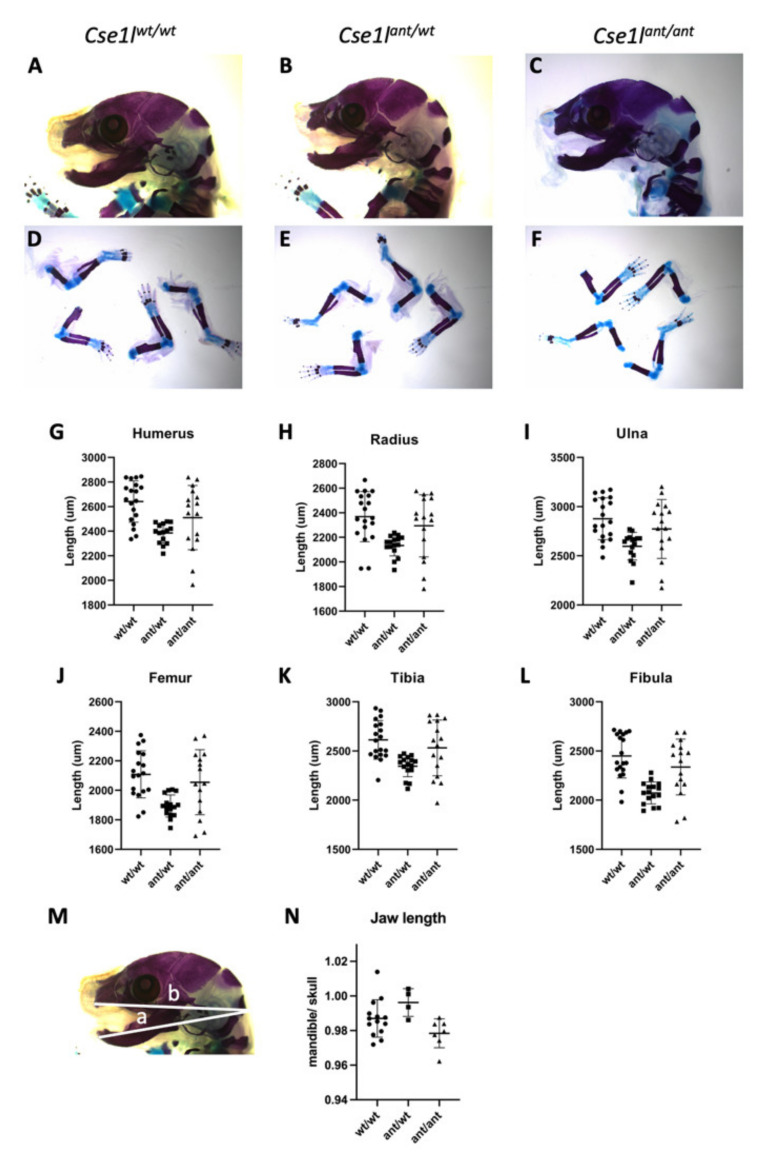
*Cse1l* mutant skeletal analysis. Wild-type *Cse1l^wt/wt^* (**A**,**D**), *Cse1l^ant/wt^* (**B**,**E**) and *Cse1l^ant/ant^* (**C**,**F**) skull and limb skeletal preparations. (**G**–**L**) Lengths shown for humerus (**G**), radius (**H**), ulna (**I**), femur (**J**), tibia (**K**), and fibula (**L**). Measurements as outlined in (**M**) are quantified and shown in (**N**).

We performed a preliminary analysis of the *Cse1l^ant/ant^* mutant skeletal system after observing some of the obvious craniofacial phenotypes. We initially assessed skeletal preparations of E18.5 mutants and littermate controls by measuring the length of their long bones (Figure 5). The humerus, radius, ulna, femur, tibia, and fibula of mutant embryos were all slightly shorter than their wild-type counterparts, but we note a wide degree of variability in this sample set (*n* = 19 *Cse1l^wt/wt^*, 16 *Cse1l^ant/wt^*, and 16 *Cse1l^ant/ant^*). Interestingly, all of these bones were markedly shorter in the *Cse1l^ant/wt^* heterozygotes than either wild-type or homozygous *Cse1l^ant/ant^* mutant animals. We similarly measured the length of the mandible and analyzed as a ratio to the overall length of the skull (*n* = 14 *Cse1l^wt/wt^*, 4 *Cse1l^ant/wt^*, and 7 *Cse1l^ant/ant^*; Figure 5M,N) to differentiate between generally smaller embryos and discrete changes in skeletal lengths. We see an increase in this ratio suggesting a small overall head relative to the mandible and a small decrease in the homozygous mutant. The biological significance and mechanism of the larger, more significant changes restricted to the heterozygotes remains unclear (Table 1).

Statistical analysis is presented separately (Table 2).

**Figure 6:**
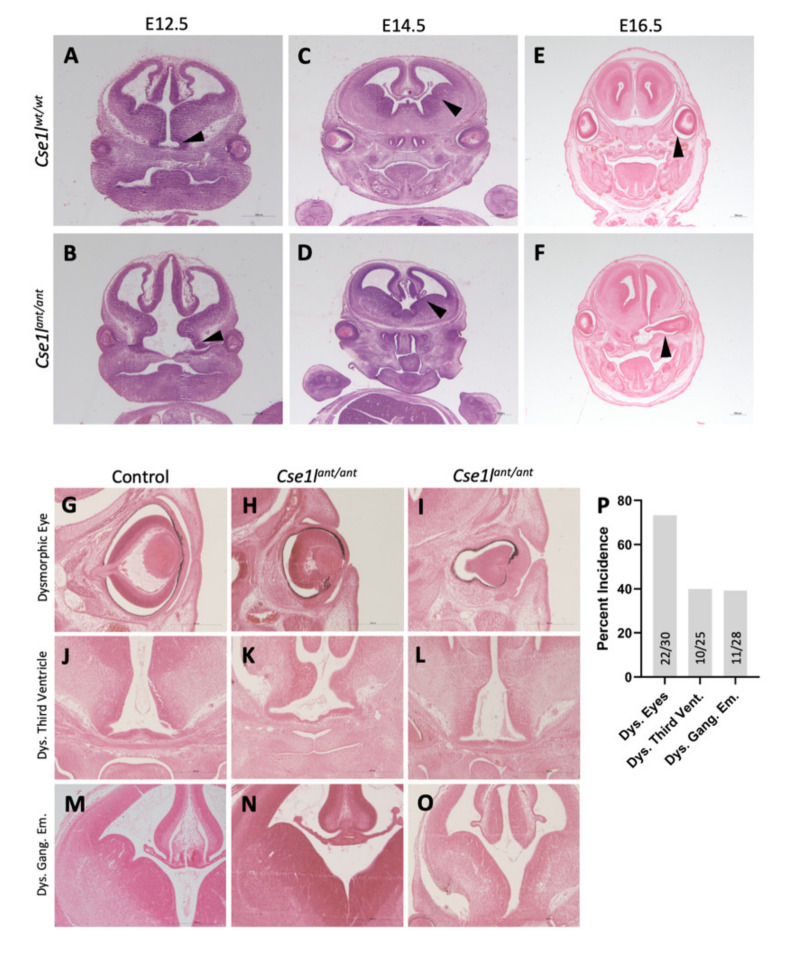
Histology of *Cse1l^ant/ant^* mutants reveals eye and brain structural abnormalities. *Cse1l^wt/wt^* at E12.5 (**A**), E14/5 (**C**) and E16.5 (**E**) and *Cse1l^ant/ant^* mutants at E12.5 (**B**), E14.5 (**D**), and E16.5 (**F**) show a range of phenotypes quantified in (**G**). (**H**–**P**) Detailed view of ocular (**I**,**J**), third ventricle (**L**,**M**) and ganglionic eminence (**O**,**P**) *Cse1l^ant/ant^* mutant phenotypes.

We also performed a histological analysis of the developing forebrain at ages E12.5, E14.5, and E16.5. These sections displayed highly variable eye and brain structural abnormalities (Figure 6A–F). The most striking brain phenotypes included widening of the base of the third ventricle, loss of midline structure, and a loss of a distinct sulcus between the medial and lateral ganglionic eminences. Eye defects were the most commonly noted histological phenotype (Figure 6G). Retina development appeared to be largely complete, but eye morphology was grossly disturbed in mutants at all ages, sometimes presenting on a unilateral basis. In some cases, the eye was exposed to the lateral ventricles, while in other instances, the eye was completely internalized (Figure 6H–J).

### 3.5. Forebrain Patterning Is Not Consistently Perturbed in Anteater Mutants

**Figure 7:**
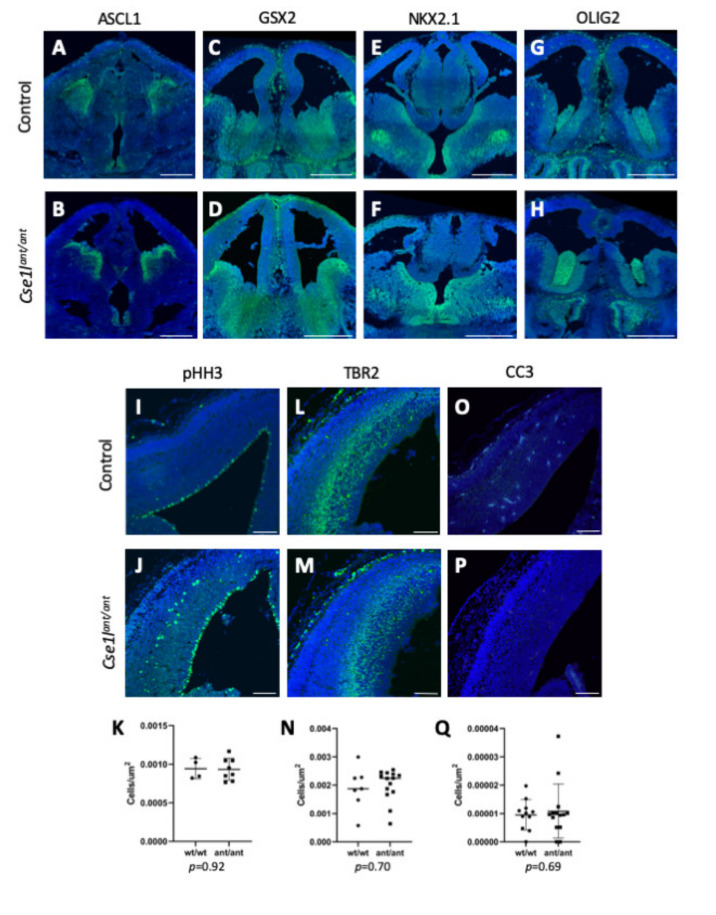
Marker analysis of forebrain shows that patterning is not disrupted perturbed in *Cse1l^ant/ant^* mutants. (**A**–**H**) Patterning markers in the developing forebrain show no difference in regionalization at E14.5 (**I**,**J**). pHH3 staining shows some mislocalized proliferating cells in mutant cortex. (**L**,**M**) Mutants show a slight decrease in TBR2 staining (**O**,**P**) and no increase in CC3 staining. Quantification of pHH3 (**K**), TBR2 (**N**) and CC3-apoptosis data (**Q**) (*p* values are the result of unpaired *t* tests). Scale bars are 500 μm in (**A**–**H**) and 100 μm (**I**–**P**).

The wide variability of phenotypes in *Cse1l^ant/ant^* mutants presents significant challenges to any rigorous molecular mechanistic studies. However, due to the various brain phenotypes observed in the *anteater* mutants, we did analyze a number of brain patterning markers in the mutants (Figure 7). ASCL1 has been shown to play critical roles in neural progenitor specification, differentiation, and growth in the telencephalon [28]. Analysis of ASCL1 in the *anteater* mutants and wild-type controls at E12.5 and E14.5 did not reveal any drastic variation in expression, indicating that these progenitor programs were relatively unaffected (Figure 7A,B). Because the phenotypes in the *Cse1l^ant/ant^* mutant medial and lateral ganglionic eminences (MGE, LGE) were one of the most striking brain phenotypes, we analyzed GSX2 expression. GSX2 is expressed as an early patterning marker of the MGE and LGE, but is expressed most highly at the dorsal LGE [24,29]. The levels of this patterning marker also appeared unchanged among the *Cse1l^ant/ant^* mutants we observed in comparison to wild-type controls at ages E12.5 and E14.5 (Figure 7C,D). Ventral forebrain patterning also appeared affected in *Cse1l^ant/ant^* mutants, thus we investigated the expression of NKX2.1 in wild-type and mutant brains at E12.5 and E14.5 as it is required for ventral telencephalon patterning, playing an integral role in maintaining the MGE [30]. Our evaluation of NKX2.1 expression in *Cse1l^ant/ant^* mutants did not reveal a difference from the wild-type controls’ expression pattern (Figure 7E,F). Finally, we also assessed the spatial patterning of OLIG2, a marker of the ventricular zone (VZ) of the ventral telencephalon, including the VZ of the MGE [31], but did not see distinct patterning differences (Figure 7G,H). We concluded that, despite some of the terminal phenotypes, there were not consistent and highly penetrant molecular hallmarks of aberrant neural patterning in *Cse1l^ant/ant^* mutants.

We next examined neurogenesis patterns and analyzed pHH3 expression as a marker of cells actively undergoing mitosis [32,33] at both E12.5 and E14.5. Quantification of proliferating cells in just the VZ revealed no difference between wild-type and *Cse1l^ant/ant^* mutant groups at either age. However, several of the *Cse1l^ant/ant^* mutant samples presented an aberrant distribution of proliferative cells in the intermediate zone (IZ) at E14.5 compared to the appropriately VZ-restricted mitoses in the wild-type samples (Figure 7I–K). TBR2 is a marker of intermediate progenitor cells [34], and we compared the expression of TBR2 in *Cse1l^ant/ant^* mutant and wild-type control tissue. We observed no quantifiable difference at E14.5 (Figure 7L–N). We next considered the possibility of increased cell death in *Cse1l^ant/ant^* mutants but observed no difference in *CC3* expression between *Cse1l^ant/ant^* mutants and wild-type controls by immunofluorescence at E12.5 and E14.5 (Figure 7O–Q). Thus, there are signs that neural development is perturbed when levels of CSE1L protein are reduced in *Cse1l^ant/ant^* mutants, but the variability and incomplete penetrance of this allele present significant barriers to a more complete understanding of these effects.

We next took a global transcriptomic approach and performed RNA-Seq analysis of wild-type heads compared to *Cse1l^ant/ant^* mutant heads at E10.5 (*n* = 3 control and 3 mutant) with the hypothesis that an earlier molecular phenotype might be more penetrant than the histological and immunohistochemical analyses presented above. This analysis showed that *Cse1l* was indeed expressed at lower levels in mutant as compared to wild-type, but the analysis did not identify any other genes or pathways with significantly disrupted expression levels (Supplementary Figure S4 and Table S2). We suspect that this is due to the wide phenotypic spectrum of *Cse1l^ant/ant^* mutants, which did not allow a common affected gene and/or network to rise above background.

### 3.6. Cse1l Is Required for Proper Pax6 Regulation in the Eye and Brain

**Figure 8:**
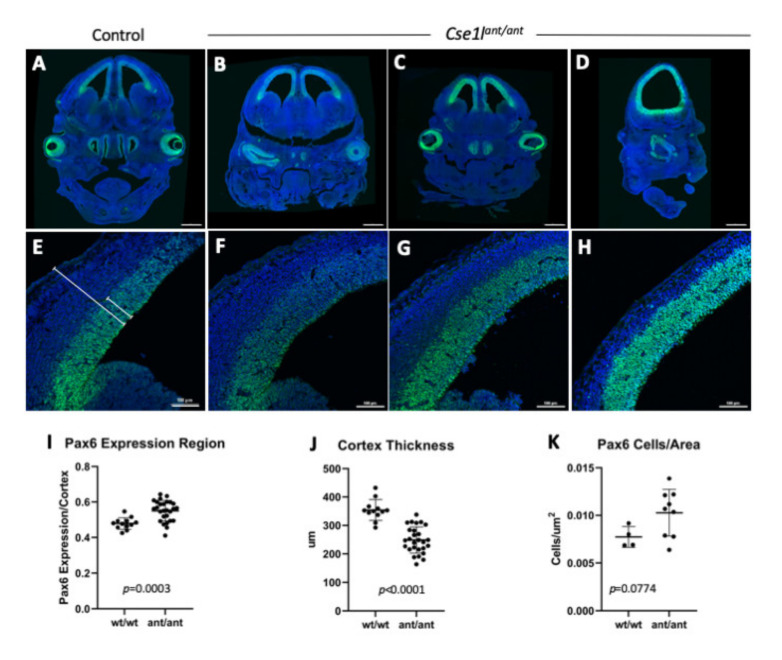
*Cse1l* is required for wild-type PAX6 expression in the brain. *Cse1l^ant/ant^* mutants as compared to *Cse1l^wt/wt^* (**A,E**) show an increase in the domain of PAX6 expression in the E14.5 cortex (**B**–**D**,**F**–**I**), as well as a decrease in cortical thickness (**J**). These combine for a higher density of PAX6-positive cells in the mutant cortex (**K**). Lines in E indicate the areas measured for cortical thickness (longer) and the PAX6 expression quantified and shown in (**I**,**J**).

*Pax6* mutations have been shown to cause developmental anomalies of the brain and eyes [35,36]. Because the most common phenotypes observed in the *Cse1l^ant/ant^* mutant included the brain and eyes, we hypothesized that changes in regulation of *Pax6* may play a role in the *Cse1l^ant/ant^* phenotype. We performed immunofluorescence staining for *Pax6* on wild-type and *Cse1l^ant/ant^* mutants at E14.5 and saw lower expression in the eyes of virtually all the *Cse1l^ant/ant^* mutants examined, as well as a generally expanded distribution of *Pax6-*positive cells in the cortex (Figure 8). Upon quantification, we measured a significant expansion of the PAX6-positive region (*p* < 0.001) as well as a significantly thinner cortex among *Cse1l^ant/ant^* mutants as compared to wild-type controls (*p* < 0.001; Figure 8J). In addition, we observed an increased density of *Pax6*-positive cells in the *Cse1l^ant/ant^* cortex (*p* = 0.078; Figure 8K).

**Figure 9:**
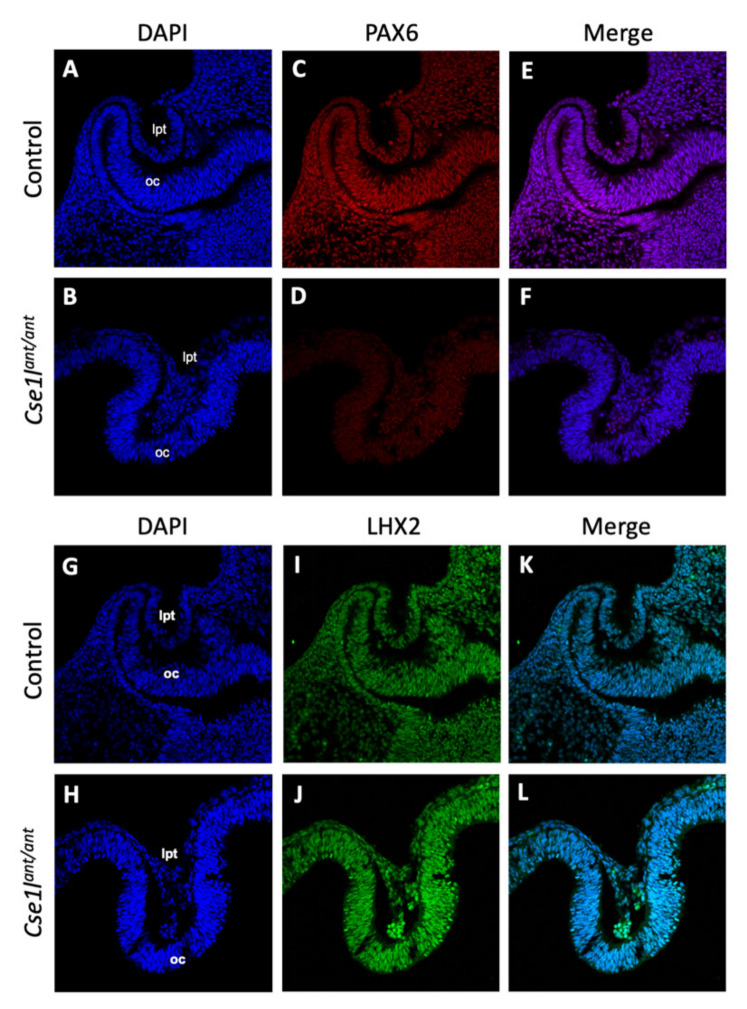
*Pax6,* but not *Lhx2* expression is decreased in *anteater* mutant eyes. Immunohistochemistry for PAX6 (**A**–**F**) and LHX2 (**G**–**L**) indicates a marked reduction in PAX6 in *Cse1l^ant/ant^* mutants (**B**–**F**), but not LHX2 (**H**–**L**).

We next sought to determine whether this impact on *Pax6* expression in the brain extended to the eyes of *Cse1l^ant/ant^* mutants. We employed immunofluorescence at E10.5 to characterize *Pax6* and *Lhx2* expression in wild-type and *Cse1l^ant/ant^* mutant eyes. *Lhx2* is a well-known gene critical for the formation of the optic cup [37]. We observed a frequent decrease in *Pax6* expression in the presumptive eye region of *Cse1l^ant/ant^* mutants, while *Lhx2* was markedly unperturbed (Figure 9).

**Figure 10:**
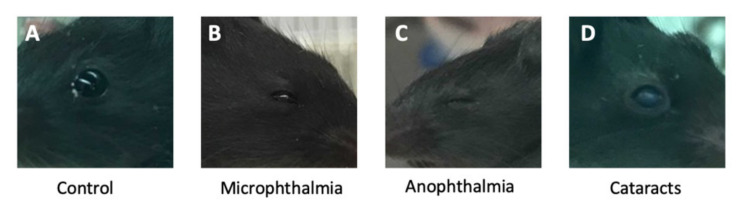
*Cse1l* CRISPR null heterozygotes may display ophthalmic abnormalities including microphthalmia (**B**), anophthalmia (**C**), and early-onset cataracts (**D**). Wild-type shown in (**A**).

Interestingly, as the eye phenotypes are the most consistent phenotype in the *Cse1l^ant/ant^* animals, we also noted that some of the heterozygous *Cse1l* CRISPR animals had congenital eye abnormalities at weaning. These animals displayed a range of conditions including microphthalmia (Figure 10A), anopthalmia (Figure 10B), and cataracts (Figure 10C).

The variability in phenotypes is so broad we considered the possibility that a genetic modifier is present in the colony. However, we performed a pedigree analysis of phenotypically similar mutants recovered from dissections separated by more than three years (e.g., Supplementary Figure S5A,C,M,O). The frequent backcrossing to C57BL/6J throughout multiple generations suggests the *anteater* phenotype is not likely due to a modifier, as it would be extremely unlikely for an unlinked modifier to segregate closely enough to *Cse1l* through these crosses to affect the phenotypes we see. The frequent backcrossing and intercrossing suggests these mice are nearly congenic. We therefore suggest the variability is more likely due to small stochastic changes in CSE1L protein function and/or level(s) in relevant cell types.

## 4. Discussion

CSE1L functions in nuclear transport as a re-exporter of importin-α, and it has been implicated in a wide range of cellular processes including cell cycle control, chromosome stability, apoptosis, and even direct gene regulation. While *Cse1l* has been shown to be critical for embryonic development, further study in organogenesis has proved elusive due to the early embryonic lethality [21]. Here, we have described the *anteater* mutant, shown that it is a novel hypomorphic allele of *Cse1l*, and used the model to demonstrate a role for *Cse1l* in mouse brain and eye formation. Upon discovering the *anteater* mutant in a forward genetic screen, we noted a decrease in mutant survival at weaning as well as a decrease in adult size, weaning weight and skull size, and embryonic long bone length. We observed a variable range of phenotypes including eye, brain, and facial dysmorphologies. The affected tissues and *Cse1l* expression patterns suggest that one particularly affected cell population is likely the neural crest. Further analysis of the phenotypes or the underlying molecular mechanisms has been significantly hampered by the wide range of phenotypes present in the mutants. The most consistent phenotype in both *Cse1l^ant/ant^* mutants and the adult *Cse1l^null/wt^* mice is an eye phenotype. This is in line with robust expression of *Cse1l* in lens development, as indicated by iSyTE analysis of the lens transcriptome and proteome. We next showed that *Pax6* is generally upregulated in *Cse1l^ant/ant^* mutant brains and that the PAX6-positive region of the cortex is expanded in the mutants, while the overall thickness of *Cse1l^ant/ant^* mutant cortex is decreased (Figure 6). However, the opposite pattern was very frequent for *Pax6* expression in the presumptive *Cse1l^ant/ant^* eye, as *Pax6* expression was clearly diminished while *Lhx2* expression remained unchanged (Figure 7). The presence of robust LHX2 expression in the presumptive *Cse1l^ant/ant^* eye tissue serves to indicate that absence/severe reduction in PAX6 is not reflective of a complete absence of eye tissue. Because of this difference in expression, we conclude that *Cse1l* plays a role in the regulation of *Pax6* in the developing brain and eye.

Because of CSE1L’s integral function in nuclear transport, it plays an important role in mediating a variety of cellular programs and signal transduction pathways. CSE1L functions as the exporter of importin-α; it has been shown in *Xenopus laevis* that disruption in levels of importin-α can have striking consequences in the developing embryo [38]. CSE1L interacts with various members of the Ras/Raf/MEK/ERK pathway, the PI3K pathway, and the cAMP pathway, serving as a hub for various signal transduction pathways [11,39,40]. Notably, CSE1L induces the phosphorylation of MITF, a gene involved in eye development [11,41]. Through MITF regulation, *Cse1l* mediates cell survival, proliferation, melanogenesis, and metastasis [42,43,44]. PAX6 and MITF have been shown to interact in a network arbitrating the counterbalanced developmental programs of retinogenesis and presumptive RPE melanogenesis [40,45,46]. These systems must be intricately synchronized to facilitate proper development of the eye. While the interaction of MITF and CSE1L is tantalizing, it does not account for the downregulation of *Pax6* upon CSE1L decrease.

Our research introduced a fascinating role for *Cse1l* in the regulation of *Pax6* in the developing brain and eyes, and we propose a mechanism to explain this regulation. The epigenetic effect of *Cse1l* on gene expression must also be considered. *Cse1l* downregulation was shown to influence the expression of a variety of transcription factors and affect the activation of silenced genes without altering their methylation status [16]. The function and regulation of *Pax6* are known to be impacted by epigenetic modification, including the gene’s methylation [47]. It is feasible that the elusive epigenetic control *Cse1l* exerts is the mechanism by which it is able to mediate *Pax6* expression in various tissues during development. This regulation of *Pax6* by *Cse1l* may explain the early onset cataracts and microphthalmia in *Cse1l^null/wt^* mice, as well as the range of eye abnormalities in the *anteater* mutants [48,49]. In the future, it will be interesting to learn what effect the downregulation of *Cse1l* will have on its various signaling pathway interactors. While the early lethality of the *Cse1l* null mouse has formerly precluded this study, the *anteater* mouse now provides an excellent means of elucidating these interactions, as we have shown that it is a functional hypomorphic allele. Further detailed characterization of retinogenesis and melanogeneis in the developing eye in the context of *Cse1l* mutations may also shed light on the complex mechanism regulating *Pax6* and MITF interactions. In addition, the development of a *Cse1l* conditional mouse model will be extremely beneficial to elucidate the effect of *Cse1l* on specific tissues and should mitigate the phenotypic variability we observed in our study. Understanding *Cse1l’*s role in specific regions of the brain and face will contribute to our further understanding of the fine-tuned control of gene expression by nuclear transport regulation.

## Figures and Tables

**Table 1:** Survival of *anteater* mutants.

*Cse1l^ant/wt^* x *Cse1l^ant/wt^*	*wt/wt*	*ant/wt*	*ant/ant* (% of Expected)	Total	Chi^2^ *p* Value
E10.5	10	27	12 (96%)	50	0.712
E12.5	23	30	14 (84%)	67	0.207
E14.5	50	125	41 (76%)	216	0.047
E16.5	8	16	7 (90%)	31	0.953
E17.5, 18.5	9	18	6 (73%)	33	0.664
P28	70	133	6 (11%)	209	1.30 × 10^−12^

**Table 2:** Statistical Analysis of Skeletal Elements.

	Genotype	Mean (st. dev)	ANOVA F Statistic (*p* Value)	Comparison	Tukey’s Multiple Comparison-Adjusted *p* Value *
**Humerus**	*wt/wt*	**2641 (168.3)**	**8.01 (0.001)**	**wt vs. ant/wt**	**0.001**
	*ant/wt*	2385 (80.94)		ant/wt vs. ant/ant	0.156
	*ant/ant*	2511 (261.2)		wt vs. ant/ant	0.108
**Radius**	*wt/wt*	**2369 (204.2)**	**6.56 (0.003)**	**wt vs. ant/wt**	**0.002**
	*ant/wt*	2133 (83.8)		**ant/wt vs. ant/ant**	0.498
	*ant/ant*	2294 (252.3)		wt vs. ant/ant	0.059
**Ulna**	*wt/wt*	**2877 (213.4)**	**6.71 (0.003)**	**wt vs. ant/wt**	**0.002**
	*ant/wt*	2597 (140.6)		ant/wt vs. ant/ant	0.083
	*ant/ant*	2773 (299.2)		wt vs. ant/ant	0.369
**Femur**	*wt/wt*	**2108 (159.7)**	**7.96 (0.001)**	**wt vs. ant/wt**	**0.001**
	*ant/wt*	1894 (75.1)		ant/wt vs. ant/ant	0.021
	*ant/ant*	2055 (220.3)		wt vs. ant/ant	0.614
**Fibula**	*wt/wt*	**2450 (224.5)**	**13.26 (<0.0001)**	**wt vs. ant/wt**	**<0.0001**
	*ant/wt*	2073 (111.6)		ant/wt vs. ant/ant	0.004
	*ant/ant*	2337 (283.1)		wt vs. ant/ant	0.294
**Tibia**	*wt/wt*	**2613 (197.0)**	**7.34 (0.002)**	**wt vs. ant/wt**	**0.001**
	*ant/wt*	2346 (108.5)		ant/wt vs. ant/ant	0.486
	*ant/ant*	2531 (283.3)		wt vs. ant/ant	0.040
**Mandible/** **Skull Ratio**	*wt/wt*	**0.987 (0.011)**	**4.311 (0.026)**	**wt vs. ant/wt**	**0.243**
	*ant/wt*	0.996 (0.008)		ant/wt vs. ant/ant	0.022
	*ant/ant*	0.978 (0.008)		wt vs. ant/ant	0.168

* The mean length of each indicated bone is shown along with the statistical analysis of the differences in length between wild-type (wt/wt), *Cse1l* heterozygotes (*ant/wt*), and *Cse1l* homozygous mutants (*ant/ant*).

## Data Availability

Data is contained within the article or supplementary material.

## Supplementary Materials

The following are available online at https://www.mdpi.com/article/10.3390/jdb9030027/s1, Figure S1: A null allele of *Cse1l*; Figure S2: *Cse1l* expression in embryonic and adult mouse lens; Figure S3: *Cse1l^ant/ant^* mutants that survive are small and dysmorphic; Figure S4: RNA-Seq differential expression analysis; Figure S5: The variable *anteater* phenotype is not likely due to a modifier; Table S1: *anteater* exome analysis; Table S2: RNA-Seq analysis.
