# Supplementary material for: A Novel Mutation in Cse1l Disrupts Brain and Eye Development with Specific Effects on Pax6 Expression
Source: J Dev Biol. 2021 Jul 7;9(3):27. doi: 10.3390/jdb9030027 (PMC8293161; doi:10.3390/jdb9030027)
Supplement: Supplementary file 1 [file jdb-09-00027-s001.zip › jdb-1225196 - supplementary/jdb-1225196 - Supplementary Figures.pdf]

Journal of Developmental Biology

MDPI

Supplemental Data Figure Legends

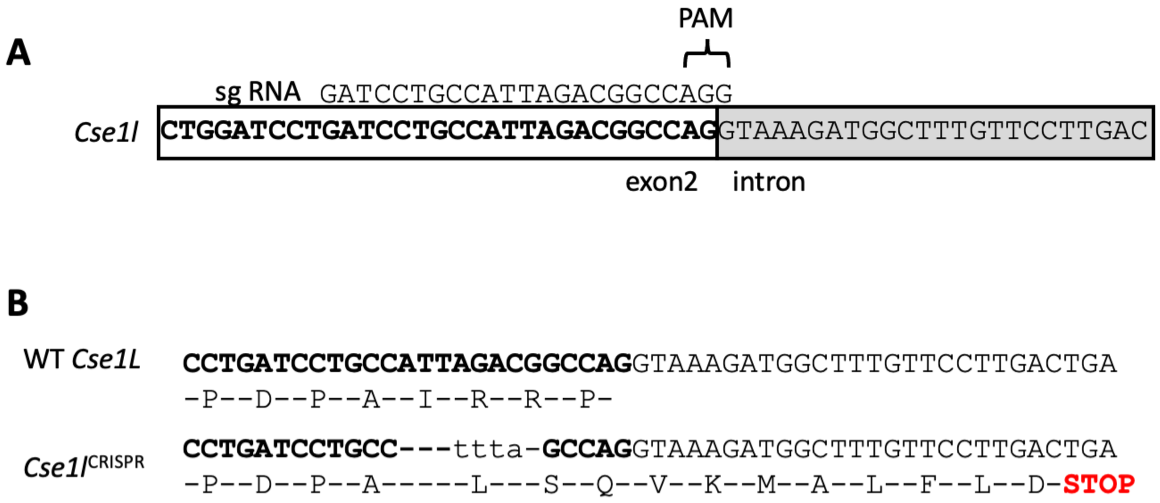

C

Cse1l ^CRISPR^ Survival at weaning	wt/wt	CRISPR/wt	CRISPR/CRISPR	Total	Chi^2 *p* value
Cse1l ^CRISPR/wt^ x *Cse1l* ^CRISPR/wt^ P28	12	16	0	28	0.004

D

Cse1l ^CRISPR/ant^ Complementation	wt/wt	CRISPR/wt	ant/wt	CRISPR/ant	Total	Chi^2 *p* value
Survival at weaning	6	4	2	0	12	0.083
Embryonic survival (E14.5-18.5)	14	13	15	0	42	0.003

Figure S1. CRISPR *Cse1l* null allele. (**A**) Guide design for *Cse1l* CRISPR null. (**B**) *Cse1l* CRISPR null sequencing reveals an 8bp deletion and a 4 bp insertion in the gene. (**C**) *Cse1l* CRISPR null embryos do not survive to P28. (**D**) *Anteater* allele fails to complement the *Cse1l* null allele.

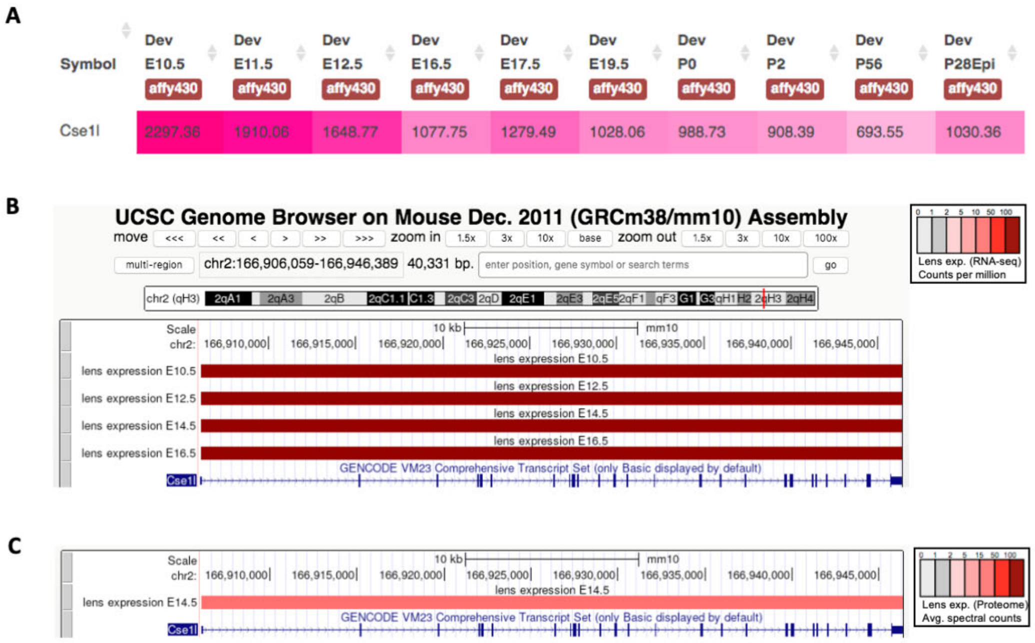

Figure S2. Cse1l expression in embryonic and adult mouse lens. iSyTE analysis based on (**A**) microarray, (**B**) RNA-seq and (**C**) proteome datasets demonstrates robust *Cse1l* expression in mouse lens. Microarray data (fluorescence signal intensity units) for whole lens at embryonic (E) stages E10.5 through E19.5 and postnatal (P) stages P0 (early postnatal) through P56 (adult) are shown. P28Epi represents microarray data on isolated lens epithelium at postnatal day 28. RNA-seq data (counts per million) for whole lens at E10.5 through E16.5 is indicated by the RNA-seq heat-map. High-throughput tandem mass spectrometry (MS/MS) proteome data (average spectral counts) by is indicated by the proteome heat-map. .

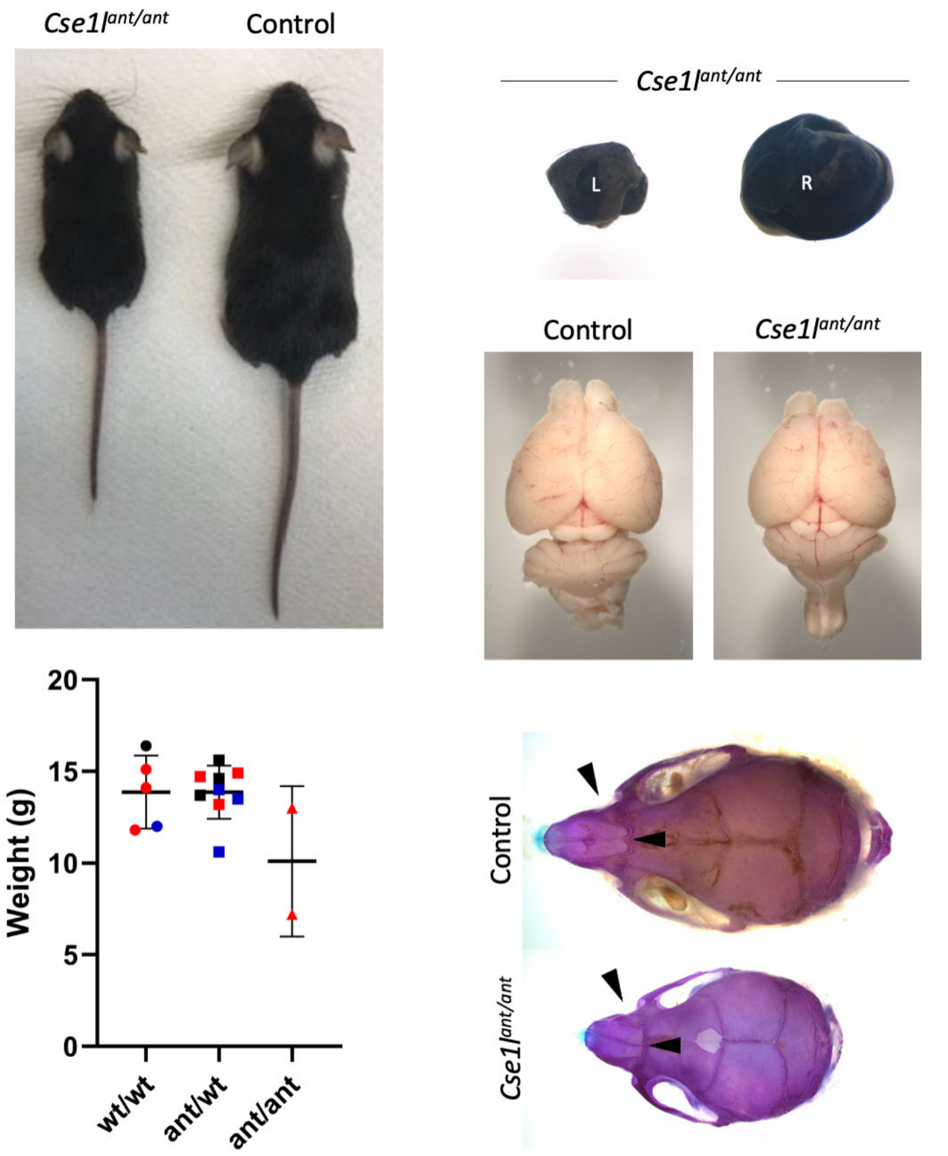

Figure S3. *Anteater* mutants that survive are small and dysmorphic. The few *anteater* mutants that survive to P28 are smaller and weigh less than their littermate controls. Some have reduced eyes but gross brain structures appear relatively normal. The skull of the P28 *anteater* mutant is smaller and dysmorphic compared to its littermate.

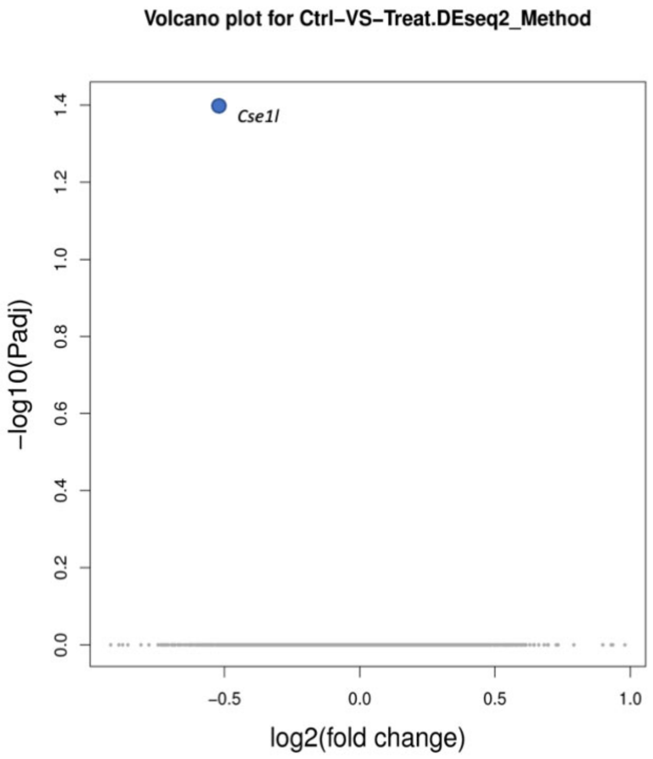

Figure S4. RNA-Seq differential expression analysis show only one gene with significant difference in expression between wild-type and mutant animals.

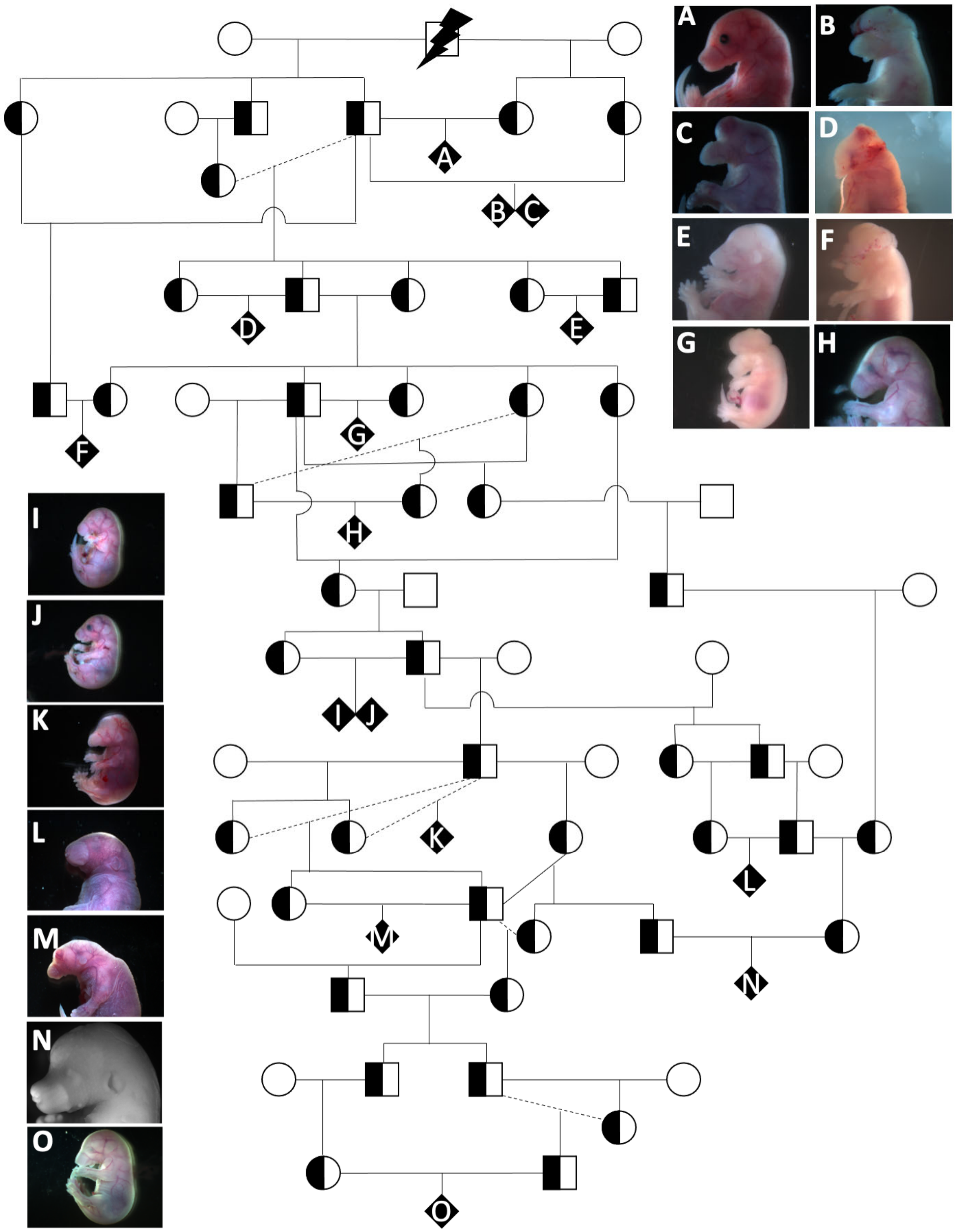

Figure S5. ***Anteater* phenotypes are not likely due to a modifier.** A selection of mutants with various phenotypes are shown with a pedigree analysis. White circles represent outcrosses to C57BL/6 mice during propagation of the colony. The initial mutagenized male is indicated with the “lightning” symbol on the top line.
